# Association between love breakup and suicidal ideation in Peruvian medical students: a cross-sectional study during the COVID-19 pandemic

**DOI:** 10.3389/fpsyt.2023.1287036

**Published:** 2024-01-29

**Authors:** Danai Valladares-Garrido, J. Pierre Zila-Velasque, Flor M. Santander-Hernández, Miguel A. Guevara-Morales, Noelia Morocho-Alburqueque, Virgilio E. Failoc-Rojas, César Johan Pereira-Victorio, Víctor J. Vera-Ponce, Darwin A. León-Figueroa, Mario J. Valladares-Garrido

**Affiliations:** ^1^Escuela de Medicina, Universidad Cesar Vallejo, Trujillo, Peru; ^2^Oficina de Salud Ocupacional, Hospital Santa Rosa, Piura, Peru; ^3^Facultad de Medicina, Universidad Nacional Daniel Alcides Carrion, Pasco, Peru; ^4^Red Latinoamericana de Medicina en la Altitud e Investigación (REDLAMAI), Pasco, Peru; ^5^Escuela de Medicina, Universidad Cesar Vallejo, Piura, Peru; ^6^Sociedad Científica de Estudiantes de Medicina, Universidad Cesar Vallejo, Piura, Peru; ^7^Escuela de Medicina Humana, Facultad de Ciencias de la Salud, Universidad Nacional de Piura, Piura, Peru; ^8^Unidad de Investigación para la Generación y Síntesis de Evidencias en Salud, Universidad San Ignacio de Loyola, Lima, Peru; ^9^Escuela de Medicina, Universidad Continental, Lima, Peru; ^10^Instituto de Investigación en Ciencias Biomédicas, Universidad Ricardo Palma, Lima, Peru; ^11^Universidad Tecnológica del Perú, Lima, Peru; ^12^Facultad de Medicina Humana, Universidad de San Martín de Porres, Chiclayo, Peru; ^13^South American Center for Education and Research in Public Health, Universidad Norbert Wiener, Lima, Peru; ^14^Oficina de Epidemiología, Hospital Regional Lambayeque, Chiclayo, Peru

**Keywords:** medical students, mental health, love, suicidal ideation, COVID-19, pandemic, Peru

## Abstract

**Objective:**

We aimed to determine the association between a major romantic breakup and suicidal ideation in medical students from three universities in Peru.

**Methods:**

A cross-sectional study was conducted during the first pandemic wave in 2021 on medical students from three universities in northern Peru. The outcome was suicidal ideation, measured with question nine of the Patient Health Questionnaire-9 (PHQ-9). Generalized Anxiety Disorder Scale-7 (GAD-7) and Insomnia Severity Index (ISI) were also used to assess mental health symptoms. The exposure was the experience of a major love breakup during the pandemic. In addition, its association with other covariates (age, sex, family members infected with COVID-19, deceased family members with COVID-19, insomnia, and anxiety, among others) was examined.

**Results and discussions:**

Out of 370 students, 19.5% reported a major love breakup during the pandemic (95%CI: 15.5–23.8), and 34.3% had suicidal ideation (95%CI: 29.4–39.4). Having a major love breakup was associated with a higher prevalence of suicidal ideation (PR: 1.49, 95%CI: 1.32–1.67, *p* < 0.001). Moderate insomnia (PR: 2.56, 95%CI: 1.70–3.87, *p* < 0.001) and anxiety symptoms (PR: 1.94, 1.10–3.44, *p* = 0.023) were also associated with suicidal ideation.

**Conclusion:**

Our study provides evidence of a significant association between a major love breakup and suicidal ideation. This finding emphasizes the need for further research to better understand this association and inform the development of effective suicide prevention policies in medical education.

## 1 Introduction

Worldwide, suicide is responsible for more than 700,000 deaths annually and is the fourth leading cause of death in young people aged 15–29 years, with a higher percentage of deaths in low- and middle-income countries (1). Suicidal ideation (SI) is considered the most important risk factor for suicide (1, 2), a public health problem that has recently begun to be studied in Latin America (3).

Medical students have a prevalence of SI from 13.7 to 33.3% (4–6). Due to the COVID-19 pandemic, not only was physical health affected (7–9), but mental health has also deteriorated significantly in various populations (10–15). Similarly, in the context of the COVID-19 pandemic, SI increased from 13.4 to 16.9%, according to a study of Mexican students (16), a result similar to a French study (17). In Peru, 17.9% of medical students had SI during the COVID-19 pandemic, which was found to be associated with a higher frequency of depression and anxiety (18). During the year 2010, it was estimated that 19.9% of 2,521 adolescent students had suicidal ideation, according to the Global School-based Student Health Survey (19). In 11,609 Peruvian adolescents, a combined prevalence of suicidal ideation of 8.5% was found after analyzing data from the Demographic and Family Health Surveys from 2013 to 2019 (20). Additionally, the development of this disease is influenced by several potential protective factors: the search for psychological help or support in their family (21), no mental health disorders and/or use of illegal substances (22), and to have stability in their love relationship (23, 24). Conversely, having depression, poor social support, less frequent conversations, and ending a stable romantic relationship all behave as aggravating risk factors for the development of SI (5, 25–27). It is known that the gender role influences the presentation of mental health disorders, evidencing very noticeable changes due to the performance of roles and the reduction of opportunities with paid jobs in women in whom affective disorders are more frequent and are increased by the presence of “macho” thinking” (28, 29), the presence of factors such as harassment and illegitimate tasks that condition the development of mental health disorders (30). Here we highlight a variable that has been focused on in groups other than medical students (5, 22–24, 31), as is the termination or breakup of a love relationship. Relationships are one of the most important social interactions for human beings, and their termination can cause physical and mental problems, such as suicidal thoughts and behaviors (32, 33). The impact of rupture is even greater in young people such as medical students (34). The breakdown of romantic relationships has increased during the pandemic due to government-imposed restrictions such as social distancing that led to staying at home (30) and actions not unrelated to Peru (35, 36). Results have been found in a higher proportion of women and health professionals (37) and even more so in the university population that has undergone curricular changes that have led to the development of mental health and stress disorders (38).

The necessity of this study stems from the urgent need to comprehensively understand the mental health challenges faced by medical students during the COVID-19 pandemic, with a specific focus on the potential impact of romantic relationship breakups. The increased incidence of romantic breakups during the pandemic, attributed to factors like social distancing and curricular changes, underscores the necessity of examining its impact on mental health (39). This is especially pertinent for medical students who navigate a unique intersection of academic and personal challenges. Given the alarming increase in suicidal ideation (SI) during the pandemic, especially among medical students (18), there is a crucial gap in our understanding of the unique factors contributing to this vulnerability. While existing studies have explored the prevalence of SI and its association with different variables (40), there is a dearth of conclusive evidence regarding the psychological ramifications of romantic breakups in medical students.

However, there is no conclusive evidence in medical students that the psychological impact of a breakup is compounded by the physical and mental exhaustion produced by the academic load (31, 38), which would lead to an increased presence of SI (1). In addition, the studies that evaluated these variables had confounding and information biases because they did not include variables that have been shown to be associated with the outcome, such as mental health disorders, and because they were measured with instruments that have not been validated in the context of the study population (24, 41, 42). Finally, the studies were conducted on smaller samples (16, 24, 43).

Therefore, in the present study, we evaluated whether love breakup influences the presence of SI, together with other variables of interest such as insomnia, a history of having a family member who died from COVID-19 and obesity, variables not included in previous studies, and that could potentially act as confounders (5, 18).

## 2 Methods

### 2.1 Study sample and setting

We conducted an analytical cross-sectional study based on a secondary analysis of a study assessing the association between Smartphone overuse and mental health disorders (44). This study was carried out among medical students in Piura between July and October 2020, a time when Peru was experiencing the first COVID-19 wave and with the restrictive measures imposed by the government to limit the increase in the number of infections. The present study aimed to evaluate whether a love breakup is associated with suicidal ideation in medical students.

The population consisted of medical students over 18 years of age who completed and accepted the informed consent form and responded to the survey. From Universidad Privada Antenor Orrego, Universidad Nacional de Piura and Universidad Cesar Vallejo. Prior to data collection, authorization was requested from the three participating universities. Subsequently, the research was conducted from July to October 2020, in the context of the first COVID-19 pandemic wave, when university higher education was providing virtual teaching in Peru. The form was designed and reviewed in Google Forms, then disseminated to the official social network groups of each year of study of the participating medical schools. The questionnaire was shared at times when the students were not in evaluations, and the approximate duration was 10 min. The questionnaire consisted of (1) informed consent, (2) socio-educational data, and (3) mental health data (PHQ-9, GAD-7), including insomnia (ISI). Finally, the data were exported from Google Forms to a database in Microsoft Excel, which underwent strict quality control prior to statistical analysis.

### 2.2 Study sample

The inclusion criteria for the primary study included students who had a cell phone with permanent access to the internet for their activities. The exclusion criteria for the primary study included self-reporting of a diagnosis of a mental health disorder (anxiety and depression). For the primary study (44), the sample consisted of 370 participants, representing a participation rate of 16.6% from the total population of 2,228 medical students across the three universities. This distribution included n1 = 151, n2 = 121, and n3 = 98 students from each respective university. The study selected participants by convenience sampling.

For this secondary analysis, the statistical power was estimated, yielding a value of 97.37%. This calculation was based on the proportion of suicidal ideation in the group without love breakup (*p*^1^ = 0.295) and the corresponding proportion in the group with love breakup (*p*^2^ = 0.541). Additionally, the respective sample sizes were considered, with n1 = 298 for the group without love breakup and n2 = 72 for the group with love breakup.

### 2.3 Instruments

Patient Health Questionnaire-9 (PHQ-9): An instrument that was validated in Peru, consisting of nine items with questions related to the presence of depressive symptoms in the last 2 weeks, evaluated on a Likert-type scale (45). The instrument has presented sensitivity and specificity values of 88 and 92%, respectively (46). In this research, the last item of the PHQ-9 was used to measure suicidal ideation (“how often have you been bothered during the past 2 weeks by thoughts that you would be better off dead or thoughts of hurting yourself in some way?”) (45). This instrument has been adapted for Peruvian university students with a Cronbach’s alpha consistency of 0.87 (47).

Generalized Anxiety Disorder Scale-7 (GAD-7): An instrument that evaluates the presence of anxiety symptoms. The instrument consists of seven items with scores ranging from zero (not at all) and three (almost every day), the total score ranges from zero to 21. The cut-off points used for the study were 0 to 4 as having no anxiety and 5 points or more as having anxiety. (48). The instrument has shown sensitivity and specificity values of 89 and 82%, respectively (48), in addition to a Cronbach’s alpha consistency of 0.89 in Peruvian students (49). This instrument has been adapted for Peruvian university students (50).

Insomnia Severity Index (ISI): An instrument that assesses the presence of insomnia symptoms by means of an instrument that evaluates seven items, an instrument that has been validated in Spanish (51). It is composed of 7 items that assess the nature, severity, and impact of insomnia, with a total score of 28 points. The cut-off points used for insomnia were 0 to 7 as the absence of pathology, 8 to 14 as mild insomnia, 15 to 21 as moderate to severe insomnia and 22 to 28 as severe insomnia. The sensitivity and specificity was 86.1 and 87.7%, respectively (52), in addition to a Cronbach’s alpha consistency of 0.84 (53). This instrument has been adapted for the Latin American population, showing high psychometric properties (54).

Sociodemographic and educational data: age in years, sex, single marital status (no, yes), obesity (no, yes; based on body mass index calculation using self-reported weight and height), self-report of having had a family member infected (no, yes) and deceased by COVID-19 (no, yes), and self-report of having suffered a serious economic problem in the last 3 months (no, yes). It is important to mention that these variables were chosen based on the literature review and the context in which the study was conducted.

### 2.4 Primary outcome

The dependent variable was suicidal ideation, defined as a student’s response to question 9 of PHQ-9. The question assesses whether respondents had thoughts that they would prefer to be dead or to harm themselves in some way. The initial responses were no days, several days, more than half of the days, and almost every day. For the analysis of this research, it was dichotomized into no and yes (several days-almost every day).

### 2.5 Secondary outcomes

The primary independent variable was major love breakup, defined as a student’s self-report of having suffered a major relationship breakup during the COVID-19 pandemic (no, yes).

Secondary independent variables were age in years, sex (female, male), single (no, yes), having obesity (no, yes), report of having had a close relative with COVID-19 (no, yes), report of having had a deceased relative with COVID-19 (no, yes), report of having suffered a serious financial problem in the past 3 months (no, yes), insomnia (no, below the threshold, moderate, severe), and anxious symptoms (no, yes).

### 2.6 Statistical analysis

The statistical analysis was performed in Stata 16.1.

For descriptive analysis, we showed absolute and relative frequencies for categorical variables. For numerical variables, we evaluated the assumption of normal distribution and then reported the best measure of central tendency and dispersion.

For the bivariate analysis, the association of interest (love breakup vs. suicidal ideation) was evaluated, as well as the rest of the categorical covariates, through the chi-square test of independence. In the case of numerical variables, the Mann–Whitney U test was useful after evaluating the assumption of normal distribution. The significance level was 5%.

For the simple and multiple regression analysis, we used generalized linear models with Poisson distribution, robust variance, and universities as groups or clusters. This allowed us to estimate prevalence ratios (PR) and 95% confidence intervals (95%CI) for the association of interest and the rest of the exposure variables. In the multiple regression analysis, confounding variables served as a model adjustment to assess the association between love breakup and suicidal ideation. Collinearity between the variables of interest was assessed with the variance inflation factor, giving an overall estimate of 2.17.

### 2.7 Ethical aspects

The primary study was approved by the Ethics Committee of the Norbert Wiener University, Lima, Peru. Code: 1516–2022. The questionnaires were anonymous, and informed consent was obtained from all participants prior to their participation in the research, in accordance with the ethical procedures established by the Ethics Committee of Norbert Wiener University (Lima, Peru). The instruments were administered after an explanation of the benefits and risks of participating in the study, after which the responses were coded, a situation that allowed us to ensure the anonymity of the participants. The ethical principles of the Declaration of Helsinki were maintained.

## 3 Results

### 3.1 Characteristics of the participants

Of the 370 students analyzed, 61.9% were male, and the median age was 20 years (19–23); 7.6% were obese, 20.5% had moderate insomnia, and 68.9% had anxious symptoms; 19.5% reported having had a major love breakup during the pandemic (95%CI: 15.55–23.87); 34.3% of the students presented suicidal ideation (95%CI: 29.49–39.41; Table 1).

**Table 1:** Characteristics of participants (*n* = 370).

Characteristics	n (%)
Age (years)*	20 (19–23)
Sex	
Male	141 (38.1)
Female	229 (61.9)
Academic year	
First	68 (18.4)
Second	72 (19.6)
Third	70 (18.9)
Fourth	68 (18.4)
Fifth	40 (10.8)
Sixth	35 (9.5)
Seventh	17 (4.6)
Single marital status	
No	363 (98.1)
Yes	7 (1.9)
Obesity	
No	342 (92.4)
Yes	28 (7.6)
Family member diagnosed with COVID-19	
No	146 (39.5)
Yes	224 (60.5)
Family member deceased due to COVID-19	
No	276 (74.6)
Yes	94 (25.4)
Financial hardship	
No	247 (66.8)
Yes	123 (33.2)
Insomnia severity index	
No	119 (32.2)
Subthreshold	169 (45.7)
Moderate	76 (20.5)
Severe	6 (1.6)
Anxiety	
No	115 (31.1)
Yes	255 (68.9)
Love breakup during pandemic due to COVID-19	
No	298 (80.5)
Yes	72 (19.5)
Suicidal ideation	
No	243 (65.7)
Yes	127 (34.3)

*Age expressed as median and p25–p75.

### 3.2 Love breakup and other factors associated with suicidal ideation in bivariate analysis

Students who reported having a major love breakup during the pandemic had a 24.7% higher frequency of suicidal ideation compared to students who did not have a love problem (54.2% vs. 29.5%; *p* < 0.001). Having anxious symptoms increased the frequency of suicidal ideation in students by 35.9% compared to those without anxiety (45.5% vs. 9.6; *p* < 0.001). Students with severe insomnia had a 53.2% higher frequency of suicidal ideation compared to those without sleep problems (66.7% vs. 13.5%; *p* = 0.001). Additionally, age (*p* = 0.003), obesity (*p* < 0.001), having a family member with COVID-19 (*p* = 0.008), having had a deceased family member with COVID-19 (*p* = 0.020) were significantly associated with having suicidal ideation in the evaluated students (Table 2).

**Table 2:** Love breakup and other factors associated with suicidal ideation, bivariate analysis.

Variables	Suicidal ideation		p*
	No (*n* = 243)	Yes (*n* = 127)	
	n (%)	n (%)	
Age (years) †**	20 (18–22)	21 (19–24)	**0.003**
Sex			0.137
Male	86 (61.0)	55 (39.0)	
Female	157 (68.6)	72 (31.4)	
Single marital status			0.631
No	4 (57.1)	3 (42.9)	
Yes	239 (65.8)	124 (34.2)	
Obesity			**<0.001**
No	234 (68.4)	108 (31.6)	
Yes	9 (32.1)	19 (67.9)	
Family member diseased by COVID-19			**0.008**
No	84 (57.5)	62 (42.5)	
Yes	159 (71.0)	65 (29.0)	
Family member died by COVID-19			**0.020**
No	172 (62.3)	104 (37.7)	
Yes	71 (75.5)	23 (24.5)	
Financial hardship			0.379
No	166 (67.2)	81 (32.8)	
Yes	77 (62.6)	46 (37.4)	
Insomnia			**0.001**
No	103 (86.6)	16 (13.4)	
Subthreshold	115 (68.1)	54 (31.9)	
Moderate	23 (30.3)	53 (69.7)	
Severe	2 (33.3)	4 (66.7)	
Anxiety			**<0.001**
No	104 (90.4)	11 (9.6)	
Yes	139 (54.5)	116 (45.5)	
Love breakup during pandemic due to COVID-19			**<0.001**
No	210 (70.5)	88 (29.5)	
Yes	33 (45.8)	39 (54.2)	

*value of *p* of categorical variables calculated with Chi-Square test. **value of *p* of categorical-numerical variables calculated with the U-test (Mann–Whitney). †Median—interquartile range. Bold numbers emphasize significant value of ps (*p* < 0.005).

### 3.3 Love breakup and other factors associated with suicidal ideation in simple and multiple regression analyses

Table 3 shows the simple and multiple regression analyses. The simple regression model showed that students with strong love breakups during the pandemic had an 83% higher prevalence of suicidal ideation (PR: 1.83; 95%CI: 1.60–2.10). This was maintained in the multiple regression model in terms of direction and magnitude: having a major romantic breakup increased the prevalence of suicidal ideation by 49% (PR: 1.49; 95%CI: 1.32–1.67).

**Table 3:** Love breakup and other factors associated with suicidal ideation, regression analysis.

Characteristics	Suicidal ideation					
	Simple regression			Multiple regression		
	PR	95%CI	p*	PR	95%CI	p*
Age (years)	1.05	1.01–1.10	**0.048**	1.03	1.01–1.05	**0.009**
Sex						
Male	Ref.			Ref.		
Female	0.81	0.55–1.18	0.272	0.89	0.80–0.99	**0.040**
Single marital status						
No	Ref.			Ref.		
Yes	0.80	0.26–2.45	0.692	1.12	0.21–6.01	0.892
Obesity						
No	Ref.			Ref.		
Yes	2.15	1.68–2.74	**<0.001**	1.25	0.79–1.97	0.350
Family member diseased by COVID-19						
No	Ref.			Ref.		
Yes	0.68	0.53–0.88	**0.003**	0.95	0.74–1.20	0.652
Family member died by COVID-19						
No	Ref.			Ref.		
Yes	0.65	0.37–1.15	0.138	0.70	0.44–1.10	0.121
Financial hardship						
No	Ref.			Ref,		
Yes	1.14	0.82–1.58	0.434	1.13	0.87–1.47	0.357
Insomnia						
No	Ref.			Ref.		
Subthreshold	2.38	1.40–4.03	0.001	1.58	0.94–2.64	0.084
Moderate	5.19	2.98–9.04	<0.001	2.56	1.70–3.87	**<0.001**
Severe	4.96	2.79–8.82	<0.001	1.93	0.86–4.37	0.112
Anxiety						
No	Ref.			Ref.		
Yes	4.76	2.76–8.20	<0.001	1.94	1.10–3.44	**0.023**
Love breakup during pandemic due to COVID-19						
No	Ref.			Ref.		
Yes	1.83	1.60–2.10	**<0.001**	1.49	1.32–1.67	**<0.001**

*Adjusted for age, sex, single marital status, obesity, family member diseased or dead by COVID-19, financial hardship, insomnia, and anxiety. Universities were set as clusters. Bold numbers emphasize significant *p* values (*p* < 0.005).

Having moderate insomnia increased 156% the prevalence of suicidal ideation (PR: 2.56; 95%CI: 1.70–3.87). Students with anxious symptoms had a 94% higher prevalence of suicidal ideation (PR: 1.10–3.44). Additionally, for each additional year of age, students had a 3% higher prevalence of suicidal ideation (PR: 1.03; 95%CI: 1.01–1.05). Female students had an 11% lower prevalence of suicidal ideation (PR: 0.89; 95%CI: 0.80–0.99; Table 3).

## 4 Discussion

### 4.1 Prevalence of suicidal ideation in medical students

We found that the prevalence of SI was 34.3% differing notably from pre-pandemic studies. In Ecuador, among medical and psychology students, severe SI was reported at 4.5%, moderate at 19.1%, and mild at 76.4%. It should be noted that the different results could be due to the use of a different instrument, such as the ISO-30 (Inventory of Suicide Orientations) (55). Similarly, Colombian medical students showed a 17.7% SI prevalence (56), while in Mexico, it was 8.7% among undergraduate and graduate students (3). In Peru, applying the MINI (Mini-International Neuropsychiatric Interview) test revealed a 11.2% SI prevalence among medical students (57), and other studies across various disciplines reported SI rates ranging from 8.9 to 35.2%, using self-developed instruments (58, 59). These results are supported by a meta-analysis that has shown that in Latin America, the prevalence of SI is 13.8%, lower than in Europe and the United States (3). Similarly, our result differs from those reported by medical students in other countries, such as Italy (4), Iran (60), and Ethiopia (5) (13.7, 17.0, and 23.7%, respectively). It should be noted that these studies were conducted in the pre-pandemic period. Moreover, the pandemic itself may have played a significant role in exacerbating mental health challenges, including SI. The unique stressors brought about by the COVID-19 crisis, such as social isolation, uncertainties about the future, and disruptions in daily life, could contribute to higher SI rates among our study population compared to pre-pandemic periods.

In the context of COVID-19, our results differ from a US study reporting 12.7% SI in undergraduates during the second wave (61) and from a study in Mexican medical students reporting 18.6% SI in the first wave (16). Likewise, it is higher than that found in medical students in Peru during the first pandemic wave, where a prevalence of 17.9% of SI was estimated (18). The disparate results could be due to the different moments of the application of the instrument since our study was at the beginning of the pandemic during the first wave, when the uncertainty and consequences in the near future caused fear and anxiety in the population, while the other study was conducted at the end of the second wave (61). At this point, we also highlight that no other Latin American study has been found in the pandemic context on the evaluation of SI, so our study adds data to the current literature.

In Peru, data from the National Institute of Mental Health indicate a 43.2% increase in monthly SI consultations during the pandemic (62). This supports the results found in our study, which could be explained by the fact that medical students were exposed to more stressors. In addition, it was conducted in a different geographical area and with a different instrument than those used in the aforementioned studies, such as the Beck Depression Instrument (BDI-II) (4), and the Depression, Anxiety and Stress Scale (DASS) (5).

### 4.2 Frequency of severe love breakup in medical students

The present study found that 19.5% of the students reported having had a major romantic breakup in the last 3 months, lower than that reported by studies conducted in the pre-pandemic context by Espinosa et al. with a frequency of 46.9% of breakups in university students of various degrees of the FESI, UNAM in Mexico, possibly due to cultural differences, stress, anxiety, and lack of management of interpersonal problems. Similarly, it has been reported that 59.6% of university students in careers other than human medicine reported a romantic breakup (63).

This difference could be explained by the fact that 66.5% of these students belonged to the psychology career, while the medical students came expressing stress, anxiety, and SI without mentioning any love breakup. Our result is different because it was carried out in the context of the pandemic; however, we did not find other studies that evaluate this variable in the context of the pandemic. The fact that at least two out of 10 students presented a strong romantic breakup during the pandemic could be explained because social distancing measures such as staying at home were established, a situation that led to the development of stress that was greater because confinement predisposes the couple to stay alone and that their problems are highlighted and cannot be solved due to the lack of social and work interactions (64).

### 4.3 Association between having had a major breakup and suicidal ideation

Students who reported having had a major love breakup had a 38% higher prevalence of SI during the pandemic. This is similar to the findings of Tan et al. (25) in Malaysian medical students, one of the significant predictors of SI was breaking up a stable romantic relationship (OR: 5.4). Furthermore, it is consistent with another study in which the prevalence of suicide was higher (17.0%) in Iranian medical students who were separated or divorced (60). On the other hand, Barajas Marquez (65) reported that university students who had had a recent love breakup presented a higher level of depression. This is consistent with what was reported prior to COVID-19 by Espinoza-Sierra et al., who stated that the main cause of university students visiting the crisis, emergency, and suicide care center (CREAS) is the breakup of a couple, followed by bereavement and relationship problems. While for McLaughlin and Gunnell (66), who collected information on deaths of university students in the United Kingdom between 2010 and 2018, it is worth noting that the study was conducted in a pre-pandemic context, the most influential factors in suicidal behavior were love breakups, failing subjects, economics and recent bereavement. This is also evidenced in a systematic review that reported that separation or love breakup represents the most important risk factor for the development of SI in young people aged 15–29 years (32). The association found differs from that reported by Kazan (32) in Australia during the pre-pandemic context, where it was reported that women especially experienced relief and benefited from ending an abusive or negative relationship. The latter study, however, was conducted in the general adult population and not in medical students like the present study (32) and explored the quality of the relationship rather than the specific event of a breakup. It is worth noting that we did not find other studies evaluating this association, particularly during the context of the COVID-19 pandemic.

This association may be intricately linked to the profound impact of partner-related challenges, which can serve as potent triggers for SI. The heightened vulnerability observed could be attributed to a cascade of factors, most notably the diminished resilience that has become increasingly evident in the context of the pandemic (67, 68). The emotional toll of love breakups, exacerbated by the isolation and uncertainties imposed by the pandemic, may contribute to a diminished capacity for emotional control. The lack of psychological support from institutions and, crucially, the corresponding family support further exacerbates the emotional distress experienced by individuals navigating through the aftermath of a significant romantic relationship dissolution (67, 68). Understanding the psychological mechanisms underlying this association necessitates delving into the intricate dynamics of coping strategies and emotional regulation. The pandemic has undoubtedly magnified the importance of resilience in the face of adversities, and the breakdown of a romantic relationship may act as a pivotal stressor, pushing individuals toward heightened SI. The absence of robust support systems, both from institutions and family, may leave individuals grappling with emotional distress without adequate avenues for coping and recovery (67, 68).

### 4.4 Other factors associated with suicidal ideation

Having insomnia was associated with an 85% higher prevalence of SI. This result is similar to that described before the pandemic by Liu et al. (69) in Chinese university students who presented 5 times the risk of suffering SI (OR: 4.98). This is consistent with that described by Khader et al. (70), who reported that university students with insomnia presented four times more SI in the pre-pandemic context. This is consistent with that described by King et al. (71), who conducted a study at a Canadian university and found a positive association between insomnia and suicidal ideation. Moreover, it is consistent with the findings of Akram et al. (72), who conducted a study in the pre-pandemic context and found a positive correlation between insomnia and suicidal ideation in American college students. This association is explained by different postulated mechanisms, such as the decrease or alteration of serotonin 5-HT receptors (an amino acid involved in the maintenance of sleep) in subjects with suicidal tendencies (73), nightmares (74) and the alteration of the hypothalamic–pituitary axis (75). However, its association remains under constant investigation, although bidirectionality has also been found because people with SI also have the subsequent development of insomnia between this association and insomnia (76).

The study found a 3% increase in the prevalence of SI for each additional year of age, aligning with Schwenk’s pre-pandemic research on US medical students. Schwenk reported a higher SI frequency in third and fourth-year students (7.9%) compared to first and second-year students (1.4%) (77). In contrast, Khader’s pre-pandemic study in Pakistani university students across different disciplines did not identify a significant positive association between the completed university year and SI (70). This observed age-SI association in our study may be influenced by the academic load, organizational challenges, and exposure to death and suffering perceptions during clinical practice.

Female students had an 11% lower SI prevalence, contrary to studies by Osama et al. (78) and Schwenk et al. (77), which linked higher SI rates in women to elevated depression levels (18.0%). This discrepancy may stem from societal expectations on women, placing a significant social burden on them to provide stability at home. This pressure is accentuated in regions with high machismo percentages, leading to increased insecurity, limited expression of opinions, and reduced capacity to fulfill responsibilities. The United Nations reports global intimate partner violence in women aged 15 to 49, ranging from 33.0 to 51% (79), further compounding emotional image-related challenges in women (80).

Having anxiety increased the probability of SI by nine times. This is similar to that reported by Xu et al. (81), who found that Chinese medical students in the first pandemic wave presented who had anxiety symptoms were more likely to have SI (OR = 1.66). In Peru, Crisol-Deza et al. (18) reported that SI was associated with a higher probability of anxiety in medical students during the first wave of the pandemic (OR: 2.01). This is consistent with that described by Asfaw et al. (5) in Ethiopian students in the pre-pandemic context, where anxiety and depression were associated with an increased likelihood of SI. We have not found a study that identifies depression as an attenuating factor for the development of SI. This association could be due to the characteristics of a university student, loneliness, social shelter, and economic limitations that increased during the context of the pandemic, as mentioned above, due to government restrictions to limit the increase in infections.

### 4.5 Limitations and strengths

Our study has important limitations. First, the cross-sectional study design does not allow us to establish causality between variables. Second, selection bias, since a convenience sample was taken, it is not possible to infer the results for the entire population of interest. Third, being a secondary data analysis study, there is an unmeasured confounding effect since potential confounders such as the level of resilience of the students and the level of family communication, which behave as predisposing factors to various mental health disorders, have not been investigated (82, 83).

However, the study presents strengths. First, to our knowledge, it is the first study to evaluate this association of variables conducted during the COVID-19 pandemic, particularly during the first wave, the most critical time in Peru (84–88). Second, it was possible to capture a broad and varied sample (years of study) of students, and it is reinforced by the stratified choice of the sample by the university, which will serve as an aid to the strategies of the corresponding institutions. Third, several validated and widely used instruments were used in the scientific field, in addition to using a timely methodology considering the mediation of intervening variables.

### 4.6 Relevance of findings in mental health

This study adds to the current literature an underexplored finding, which proposes a precedent for future research in mental health. The students surveyed show a significant prevalence of romantic breakups that might trigger suicidal ideation and suicide in the worst-case scenario. Our results could be the basis for driving possible implications at the public health level by promoting services that eliminate the detrimental aspects of love breakup through counseling and psychology sessions for the couple in university welfare centers. We believe that social and educational programs should be promoted that encourage critical reflection on SI with the aim of reversing its factors, among them the growing love breakup. We recommend that through the tutoring areas of each university, periodic evaluations should be carried out for early detection and management of suicidal behavior to develop coping strategies and solve these problems.

## 5 Conclusion

Three and two out of 10 medical students experienced SI and a major love breakup, respectively. Our main result suggests that experiencing a major love breakup might predispose to the development of SI. As secondary results, insomnia, anxiety, and being older in the university stage were associated with SI. We recommend the development of further research that clarifies the association between major love breakups and SI and that medical schools provide periodic evaluations of mental health for the timely prevention of suicide.

## Data availability statement

The dataset generated and analyzed during the current study is not publicly available because the ethics committee has not provided permission/authorization to publicly share the data, but it is available from the corresponding author upon reasonable request.

## Ethics statement

The studies involving humans were approved by Universidad Cesar Vallejo, Piura, Peru. The studies were conducted in accordance with the local legislation and institutional requirements. The participants provided their written informed consent to participate in this study.

## Author contributions

DV-G: Conceptualization, Data curation, Formal analysis, Investigation, Methodology, Project administration, Resources, Supervision, Validation, Visualization, Writing – original draft, Writing – review & editing. JZ-V: Conceptualization, Data curation, Formal analysis, Investigation, Methodology, Project administration, Resources, Supervision, Validation, Visualization, Writing – original draft, Writing – review & editing. FS-H: Conceptualization, Investigation, Methodology, Project administration, Supervision, Validation, Visualization, Writing – original draft, Writing – review & editing. MG-M: Conceptualization, Data curation, Investigation, Methodology, Project administration, Resources, Software, Validation, Visualization, Writing – original draft, Writing – review & editing. NM-A: Conceptualization, Data curation, Investigation, Methodology, Project administration, Resources, Software, Supervision, Validation, Visualization, Writing – original draft, Writing – review & editing. VF-R: Conceptualization, Data curation, Formal analysis, Investigation, Methodology, Project administration, Software, Supervision, Validation, Visualization, Writing – original draft, Writing – review & editing. CP-V: Conceptualization, Data curation, Investigation, Methodology, Project administration, Software, Supervision, Validation, Visualization, Writing – original draft, Writing – review & editing. VV-P: Conceptualization, Investigation, Methodology, Project administration, Software, Supervision, Validation, Visualization, Writing – original draft, Writing – review & editing. DL-F: Conceptualization, Formal analysis, Investigation, Methodology, Resources, Software, Supervision, Validation, Visualization, Writing – original draft, Writing – review & editing. MV-G: Conceptualization, Data curation, Formal analysis, Investigation, Methodology, Project administration, Resources, Software, Supervision, Validation, Visualization, Writing – original draft, Writing – review & editing.
